# A Label-Free Immunosensor Based on Graphene Oxide/Fe_3_O_4_/Prussian Blue Nanocomposites for the Electrochemical Determination of HBsAg

**DOI:** 10.3390/bios10030024

**Published:** 2020-03-14

**Authors:** Shanshan Wei, Haolin Xiao, Liangli Cao, Zhencheng Chen

**Affiliations:** 1 College of Electronic Engineering and Automation, Guilin University of Electronic Technology, Guilin 541004, China; 1708404001@mails.guet.edu.cn (S.W.); 19081001010@mails.guet.edu.cn (H.X.); 2 College of Life and Environmental Sciences, Guilin University of Electronic Technology, Guilin 541004, China

**Keywords:** hepatitis B surface antigen, electrochemical immunosensor, screen-printed electrode, graphene oxide, prussian blue

## Abstract

In this article, a highly sensitive label-free immunosensor based on a graphene oxide (GO)/Fe_3_O_4_/Prussian blue (PB) nanocomposite modified electrode was developed for the determination of human hepatitis B surface antigen (HBsAg). In this electrochemical immunoassay system, PB was used as a redox probe, while GO/Fe_3_O_4_/PB nanocomposites and AuNPs were prepared and coated on screen-printed electrodes to enhance the detection sensitivity and to immobilize the hepatitis B surface antibody (HBsAb). The immunosensor was fabricated based on the principle that the decrease in peak currents of PB is proportional to the concentration of HBsAg captured on the modified immunosensor. The experimental results revealed that the immunosensor exhibited a sensitive response to HBsAg in the range of 0.5 pg·mL^−1^ to 200 ng·mL^−1^, and with a low detection limit of 0.166 pg·mL^−1^ (S/N = 3). Furthermore, the proposed immunosensor was used to detect several clinical serum samples with acceptable results, and it also showed good reproducibility, selectivity and stability, which may have a promising potential application in clinical immunoassays.

## 1. Introduction

Recently, the hepatitis B virus (HBV) has become a leading cause of death worldwide [1]. Obviously, the earlier the diagnosis, the more treatment options are available and the greater the possibility of healing. Therefore, the highly sensitive detection of viruses in human serums is considered to be a key point in the processes of treatment of HBV [2,3]. Furthermore, once you have been infected with chronic hepatitis B and this is not been arrested in a timely manner and effectively, it will further evolve into acute hepatitis, chronic hepatitis and cirrhosis, and will even lead to serious complications such as liver cancer, eventually leading to death [4]. Therefore, the prevention of hepatitis has gradually become an important public health problem all over the world [5].

HBsAg is one of the main markers of HBV infection, and it usually occurs after being infected for 1–2 weeks [6]. Many methods have been exploited to detect HBsAg in the past few decades [7], including time-resolved fluoroimmunossay (TRFIA), electro-chemiluminescence immunoassay (ECLIA), gold immunochromatography assay (GICA), ELISA, etc. [8,9,10]. In particular, the ELISA test has since been seen as the “gold standard” for comparison against all newly developed immunoassays and immunosensors [11]. However, the methods above usually need expensive instruments, skilled operation and strict culture conditions, but have low sensitivity and a narrow range [12]. In contrast with this, as an innovation for conventional determination methods, electrochemical immunosensors are a kind of analytical method that combines electrochemical sensors with immunoassays [13]. Immunoassays make specific and sensitive measurements of target analytes by harnessing the high specificity of the antigen–antibody interaction [14]. Therefore, electrochemical immunosensors have attracted increasing attention in recent years due to their stable operation, convenient use, high precision and satisfactory effectiveness in practical application, etc. [15]. Accordingly, the electrochemical immunoassay is expected to become an ideal strategy among various measurement techniques for HBsAg in the future.

Moreover, electrochemical immunosensors can be classified into two types: label-free (direct assay) and labeled (brief assay) [16]. The labeled immunosensor is used to label the analyte target before detection, and monitor the immunoassay response by quantifying the labeled product. However this type of sensor’s non-specific response is small, and the detection process is complex [17]. The label-free electrochemical immunosensor vastly simplifies the preparation and operation procedures by directly measuring the physical and chemical changes during the formation of antigen–antibody complexes [18]. Based on those, label-free electrochemical immunosensors have been developed rapidly in recent years, and the further exploration of electrochemical immunosensors still has huge space for development and tremendous development potential [19].

Signal amplification and antibody immobilization are the crucial steps in the design and fabrication of highly sensitive electrochemical immunosensors [20]. Thereby, searching for ideal materials for immobilizing identifiable redox probes as trace labels is another important issue in developing a successful immunosensor [21]. Meanwhile, profound advances in nanotechnology and nanomaterials have offered powerful tools for the design of electrochemical immunosensors [22,23]. Graphene is a new, two-dimensional carbon nanomaterial, and it has promising application prospects in energy, materials science and biomedicine [24,25]. Graphene oxide (GO) is the oxidation product of graphene, and its oxygen-containing functional groups are chemically active [26]. Therefore, the special structural characteristics of GO have proved to be a promising material in designing and preparing electroactive nanocomposites, due to advantages such as an impressive surface area, high conductivity, anti-toxicity and good electron mobility [27,28,29]. Due to the unique crystalline forms and the morphologies of Fe_3_O_4_ nanoparticles (NPs), it exhibits unique physical and chemical properties. Under the influence of the nano-effect, Fe_3_O_4_ nanomaterials with different morphologies have different properties, which makes it possible to prepare some new functional materials with special performance requirements [30]. They are widely used in electromagnetics, the chemical industry, catalysis, sensors, acoustics, medicine, environmental protection and other related fields. In particular, Fe_3_O_4_ NPs are widely used in the field of electrochemical immunosensors due to the excellent catalytic performance, impeccable magnetism, good biocompatibility and large specific surface area [31,32,33]. Prussian blue has great potential in many fields and has been widely used in electrocatalysis, biosensors, chemical sensors, rechargeable batteries and electroanalytical chemistry [34]. Therefore, PB is a well-studied material that has been extensively studied in the field of electrochemical sensors and biosensors [35,36]. In addition, there are some research works which have shown that metal NPs can also can generatse a synergistic effect with GO, such as AuNPs [37] and PtNPs [38]. Normally, AuNPs could improve electrical conductivity and provide more active sites for the binding of antibodies due to their excellent physicochemical properties [39,40]. In addition, AuNPs have been widely used to improve signal intensity [41,42].

In this work, a novel label-free electrochemical immunosensor based on redox-active conductive PB/Fe_3_O_4_/GO nanocomposites and AuNPs was constructed for the detection of HBsAg. A stable PB/Fe_3_O_4_/GO composite with electrocatalytic activity was first coated on the carbon working electrode, while PB was used as the redox probe. Subsequently, AuNPs were attached onto the modified electrode by electrodeposition, which offered an interface for HBsAb immobilization. The principle of the proposed immunosensor was based on the fact that the decrease in the peak currents of PB is proportional to the quantity of HBsAg captured on the modified immunosensor. Furthermore, the proposed method can be employed to detect HBsAg in real human serum samples with satisfactory results, which provides promising potential applications in clinical immunoassays.

## 2. Materials and Methods

### 2.1. Materials and Reagents

Graphene oxide (GO) was purchased form Xianfeng Nano Materials Tech Co. Ltd. (Nanjing, China). Ethylene glycol (CH_2_OH_2_)_2_, ethanol (C_2_H_6_O), ferric chloride (FeCl_3_.6H_2_O), sodium acetate trihydrate (CH_3_COONa) and hydrochloric acid (HCl) were purchased from Xilong Scientific Company (Guangdong, China). Potassium ferricyanide (K_3_Fe (CN)_6_), L-cysteine and cholesterol were purchased from Aladdin Company (Shanghai, China). Chloroauricacid (HAuCl_4_∙4H_2_O), bovine serum albumin (BSA), carcinoembryonic (CEA), A-fetoprotein (AFP), and human serum albumin (HSA) were purchased from Sangon Biotech (Shanghai, China). Hepatitis B surface antibody and hepatitis B surface antigen were purchased from Huayang Zhenglong Biochem. Lab. (Chengdu, China). SPEs were purchased from Nanjing Yunyou Biotechnology Co, Ltd. (Nanjing, China), as well as the bare SPE (working electrode: carbon; counter electrode: carbon; reference electrode: silver/silver chloride). All other chemicals employed were of analytical grade. Double-distilled water was used in all work.

### 2.2. Apparatus

All electrochemical measurements were carried out on a CHI660D electrochemical Workstation (Chenhua Instrument Co, Shanghai, China). Constant temperature Biochemical Culture was carried out on a BSD-100 (Shanghaibo xunshiye Company, Shanghai, China). A conventional three-electrode system was used for all electrochemical measurements and a bare SPE or modified SPE was served as the measurement electrode. The pH measurements were carried out on a PHS-3E (Shanghai INESA Scientific Instrument CO. Ltd., Shanghai, China). Centrifugal processes were all accomplished by using the high-speed freezing centrifuge TGL-20M (Hunan Xiangyi Development Co. Ltd., Yiyang, China).

### 2.3. Preparation of Fe_3_O_4_/GO Nanocomposites

Fe_3_O_4_/GO nanocomposites were synthesized based on the previous reported method with a little modification [43,44]. Briefly, 15 mg GO was ultrasonic dispersed in 75 mL (CH_2_OH_2_)_2_ for 2.5 h. Afterwards, 0.81 g FeCl_3_ was added to the above GO aqueous solution and dissolved thoroughly. Then, 1.23 g CH_3_COONa was added to the mixture and stirred vigorously for 30 min, until the color changed from black to yellow brown. The obtained mixture was moved into a Teflon-lined stainless-steel autoclave and heated to 180 °C for 11 h. After it was cooled to room temperature, the obtained Fe_3_O_4_/GO nanocomposites were isolated in the magnetic field and washed several times with ethanol and ultrapure water. Finally, the products were dried in a vacuum at 60 °C for 12 h.

### 2.4. Preparation of PB/Fe_3_O_4_/GO Nanocomposites

A total of 2 mg Fe_3_O_4_/GO nanocomposites were dispersed homogeneously in 2 mL ultrapure water by sonication. Then, 2 mL of the above aqueous solution was added into a 2 mL aqueous solution (pH 1.5, adjusted with HCl) containing 15 mmol L^−1^ K_3_Fe (CN)_6_ and 15 mmol L^−1^ FeCl_3_∙6H_2_O. After vigorously stirring for 5 h, the color changed from yellow brown to dark cyan, which indicated that GO/Fe_3_O_4_ nanocomposites were completely synthesized. The final mixture was separated by a magnet and washed several times and then dispersed in 2 mL ultrapure water.

### 2.5. Fabrication of the Immunosensor

In total, 1.5 μL GO/Fe_3_O_4_/PB of the nanocomposites (1 mg/mL) were dropped onto the surface of the SPE and dried at a constant temperature in a biochemical incubator (25 °C). The modified SPE was immersed in 50 μL (0.5 mM) HAuCl_4_, which was performed by using the electrochemical workstation to complete the electrodeposition system at a potential of −0.5 V for 180 s. A total of 5.0 μL HBsAb (0.25 mg/mL) was dropped onto the modified electrode and stored at 4 °C for 12 h. For the further fabrication of the immunosensor, it was blocked through incubation in 10 μL 1% BSA for 30 min to avoid possible nonspecific adsorption. After each step, the fabricated SPE was thoroughly cleaned with PBS and dried at room temperature prior to use.

### 2.6. Electrochemical Measurements

In total, 5 μL standard HBsAg solution at different concentrations was dipped on the proposed immunosensor and incubated at 25 °C for 40 min, followed by washing with PBS buffer. After that, the electrochemical measurements were performed in 50 μL PBS (0.01 M, PH = 7.4) by cyclic voltammetry (CV), differential pulse voltammetry (DPV) and electrochemical impedance spectroscopy (EIS). The change in the peak currents was proportional to the concentrations of HBsAg captured on the modified electrode immunosensor.

## 3. Results and Discussion

### 3.1. Electrochemical Characterization of the Immunosensor

**Figure 1:**
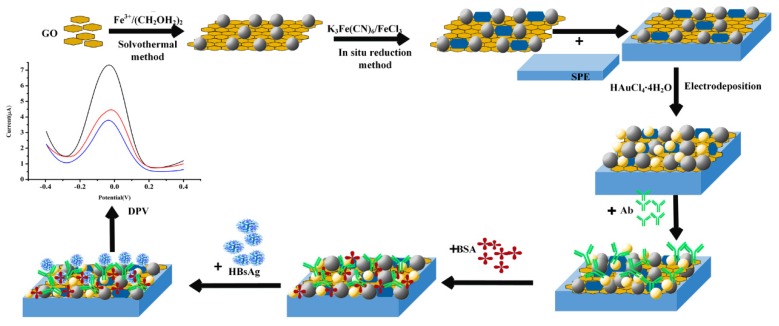
The schematic illustration for fabrication of the electrochemical immunosensor.

Figure 1 shows the fabrication procedure of the immunosensor. For the determination of HBsAg, GO/Fe_3_O_4_ nanocomposites were synthesized in the first step. As illustrated in Figure 1, Fe_3_O_4_ NPs were grown on the GO surface through the solvothermal method, while PB were attached onto the GO/Fe_3_O_4_ surface by an in situ reduction method. A stable PB/Fe_3_O_4_/GO composite with electrocatalytic activity was first coated on the carbon working electrode, while PB was used as redox probe. Subsequently, AuNPs were attached onto the modified electrode by electrodeposition, which offered an interface for HBsAb immobilization.

**Figure 2:**
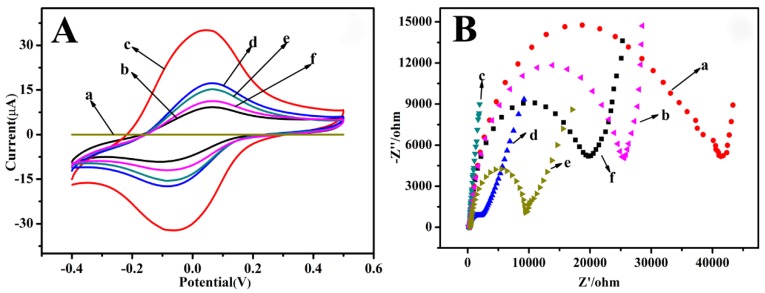
(**A**) The cyclic voltammetry (CV) of different modified electrodes in PBS. Scan rate, 100 mV/s; (**a**) bare SPE, (**b**) GO/Fe_3_O_4_/PB, (**c**) GO/Fe_3_O_4_/PB@AuNPs, (**d**) GO/Fe_3_O_4_/PB@AuNPs/HBsAb, (**e**) GO/Fe_3_O_4_/PB @AuNPs/HBsAb/BSA, and (**f**) GO/Fe_3_O_4_/PB@AuNPs/HBsAb/BSA/HBsAg. (**B**) The electrochemical impedance spectroscopy (EIS) of different electrodes in Fe(CN)_6_^3−/4−^; Init E(V): 0.24 V; frequency range: 0.1–10 KHz; Amplite: 5 mV; (**a**) bare SPE, (**b**) GO/Fe_3_O_4_/PB, (**c**) GO/Fe_3_O_4_/PB@AuNPs, (**d**) GO/Fe_3_O_4_/PB@AuNPs/HBsAb, (**e**) GO/Fe_3_O_4_/PB@AuNPs/HBsAb/BSA, and (**f**) GO/Fe_3_O_4_/PB@ AuNPs/HBsAb/BSA/HBsAg.

CV is an effective and convenient technique for probing the fabrication process of the modified electrode surface. Here, CV was used to further characterize the stepwise assembly process of the modified electrode. As shown in Figure 2A, no obvious redox peaks could be observed at the bare electrode (curve a). Subsequently, on the GO/Fe_3_O_4_/PB modified electrode, a couple of clear and symmetric redox peak at −0.048 V and −0.071 V (curve b) could be observed. The background current of the GO/Fe_3_O_4_/PB modified electrode was greater than the bare electrode, exhibiting the efficient redox activity and excellent conductivity of the GO/Fe_3_O_4_/PB nanocomposite. After HAuCl_4_ was electrodeposited onto the GO/Fe_3_O_4_/PB composite film, the peak current was further increased, which was because AuNPs could increase the electron-transfer efficiency (curve c). Furthermore, a large amount of AuNPs had strong adsorption capacity to protein and can bond with HBsAb molecule to form AuNPs–Ab nanocomposites. After the modified electrode was incubated with HBsAb, there was an obvious decrease of the current response (curve d), suggesting that HBsAb was successfully immobilized on the electrode. Then, the peak currents further decreased as BSA was employed to block possible remaining active sites (curve e). Particularly, the peak current gradually decreased after the immunosensors were incubated in HBsAg solution as the immunocomplex was formed (curve f). After this, HBsAb molecules were combined with the HBsAg molecules, which acted as the insulating protein layers on the electrode retarding the electron transfer. Especially, the decrease of the peak current was related to the amount of HBsAg captured on the modified electrode surface.

As EIS can effectively probe the electron transfer kinetics at the electrode surface, it was also used here to characterize the stepwise assembly of the immunosensor. The impedance spectra included a semicircle portion and a linear portion. At higher frequencies, the semicircle portion corresponded to the electron-transfer limited process, and at lower frequencies, the linear portion represented the diffusion-limited process. Figure 2B shows the EIS of the stepwise modification processes performed in 5.0 mM potassium ferricyanide. Compared with the bare SPE (curve a), a greatly lower resistance was obtained after GO/Fe_3_O_4_/PB was modified on the electrode, implying that GO/Fe_3_O_4_/PB nanocomposites have excellent electric conduction and could accelerate the electron transfer in to some degree (curve b). Furthermore, the GO/Fe_3_O_4_/PB @AuNPs showed a much lower resistance than the previous example, indicating that the electrodeposited AuNPs were highly beneficial to the electron transfer (curve c). In contrast, after HBsAb was immobilized on GO/Fe_3_O_4_/PB@AuNPs, the resistance obviously increased (curve d), suggesting that HBsAb was successfully immobilized on the surface and blocked the electron transport between the redox probe and electrode. Then the resistance increased again when BSA was immobilized onto the immunosensor (curve e). Finally, when HBsAg interacted with the proposed immunosensor, an increase of resistance was observed (curve f), suggesting the effective specific recognition between antibodies and antigens. This was consistent with the fact that the hydrophobic layer of protein insulates the conductive support and hinders the interfacial electron transfer. These results were in accordance with the CV measurements, demonstrating the successful construction of a GO/Fe_3_O_4_/PB-based immunosensor for HBsAg detection.

**Figure 3:**
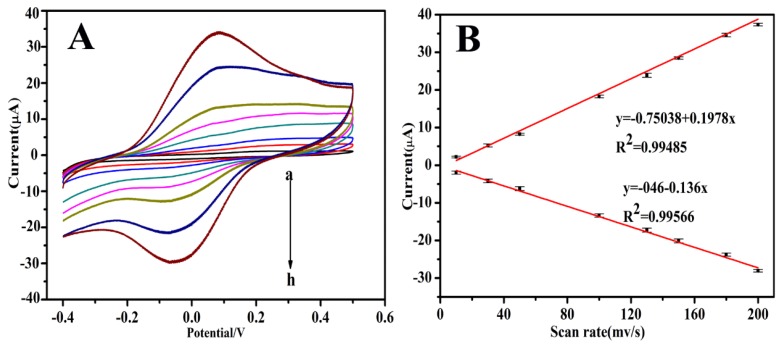
(**A**) CV of the immunosensor at different scan rates (from a–h): 10, 30, 50, 100, 130, 150, 180, and 200 mV s^−1^ in PBS solution (pH 7.4). (**B**) The linear relationship between the peak currents and the scan rate.

Valuable information involving the electrochemical mechanism can usually be obtained from the relationship between the peak current and scan rate. Here, the CVs of the modified immunosensors in 0.01M PBS (pH 7.4) at different scan rates are shown in Figure 3. It can be observed that both the anodic and cathodic peak currents were directly proportional to the scan rates in the range of 10–200 mV s^−1^. Additionally, linear relationships with good correlation coefficients were observed between the peak currents and scan rates, suggesting that the electrochemical reaction on the modified electrode is an adsorption-controlled redox process.

### 3.2. Characterization of the Modified SPEs

**Figure 4:**
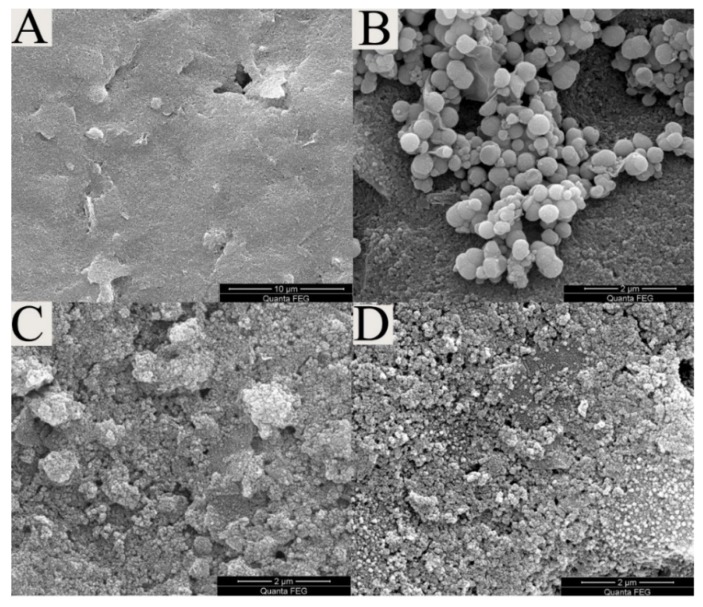
SEM images of (**A**) bare SPE, (**B**) GO/Fe_3_O_4_ nanocomposites, (**C**) GO/Fe_3_O_4_/PB nanocomposites, and (**D**) GO/Fe_3_O_4_/PB@AuNPs nanocomposites.

Figure 4A–D shows the typical scanning electron microscope (SEM) images of the bare SPE, as well as the GO/Fe_3_O_4_, GO/Fe_3_O_4_/PB and GO/Fe_3_O_4_/PB @AuNPs modified SPEs. It can be seen from Figure 4A that the SPE shows a relatively smooth surface with a few small flack-like projections. Figure 4B shows regular, compact pellets combined with lamellar folds, indicating the successful immobilization of GO/Fe_3_O_4_, and the scraggy surface can provide a large surface area for loading nanoparticles. In Figure 4C, the complicated structure of the modified SPE was observed to contain dense fine particle, indicating the successful formation of the GO/Fe_3_O_4_/PB nanocomposite film. The SEM analysis (Figure 4D) shows a good amount of granular matter, which indicated that a large number of AuNPs were immobilized onto the surface of the modified electrode. Because the structure gave a high specific surface area, it facilitates antibody attachment.

**Figure 5:**
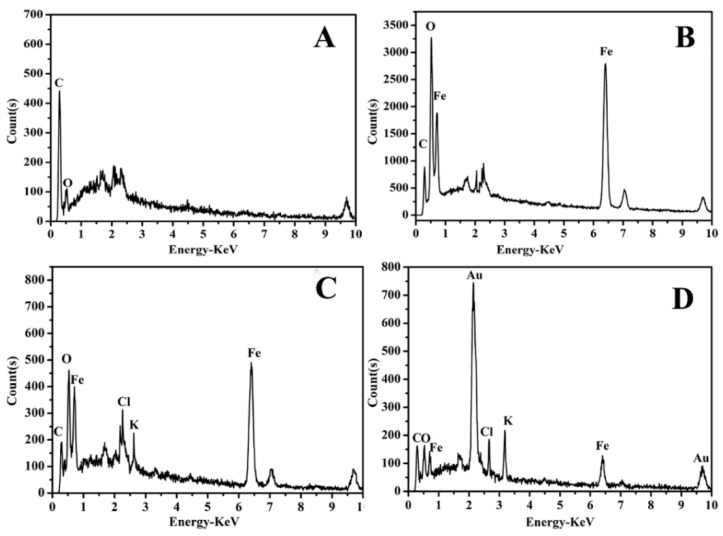
EDS spectra of bare SPE (**A**), GO/Fe_3_O_4_ (**B**), GO/Fe_3_O_4_/PB (**C**), and GO/Fe_3_O_4_/PB@Au (**D**).

Energy Dispersive Spectrometer (EDS) characterization was employed to further analyze the nanocomposites. As shown in Figure 5A, the fully scanned spectra demonstrated the existence of C and O elements in bare SPE. After the deposition of GO/Fe_3_O_4_ nanocomposites, two sharp signals related to Fe at about 0.71 KeV and 6.39 KeV could be observed (curve b). In curve c, the signals of Cl and K appeared, which came from PB nanoparticles. As shown in Figure 5D, two signals appear in the image at about 2.15 KeV and 9.7 KeV, which are clearly indicating that AuNPs attached onto the surface of the GO/Fe_3_O_4_/PB nanocomposites.

### 3.3. Optimization of Experimental Condition

To provide an optimal electrochemical experimental environment, factors include including the incubation time and concentration of HBsAb, which may affect the performance of the immunosensor, should be optimized.

**Figure 6:**
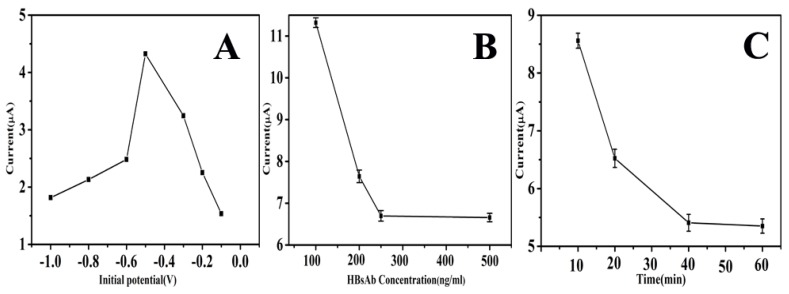
Effect of working potential on the electrodeposition of HAuCl_4_ (**A**); and effects of concentration of HBsAb (**B**) and incubation time (**C**) on the detection of HBsAg.

AuNPs have been widely applied in the construction of electrochemical biosensors, due to their excellent biocompatibility and conductivity. Therefore, it is essential to study the effect of AuNPs for the enhancement of charge transfer. The working potential of −1–0.1 V was selected for the electrodeposition of HAuCl_4_. As presented in Figure 6A, the peak current increased from −1 V to −0.5 V, achieving a maximum at −0.5 V, indicating that the optimal work potential of electrodeposition was −0.5 V.

The quantity of immobilized HBsAb is a crucial parameter in the construction of immunosensors. As shown in Figure 6B, the reduction peak current decreased with the increased HBsAb concentration, and the downtrend approximately leveled off when the concentration increased from 250 to 500 ng/mL. Therefore, 250 ng/mL was selected as the optimum incubation concentration for HBsAb in following experiments.

Incubation time also plays an important role in analyzing the performance of the immunosensor. In this experiment, the incubation time was investigated in the range of 20–60 min. As shown in Figure 6C, the results showed that the response current of the immunosensor rapidly decreased when the incubation time was increased from 20 to 40 min. When incubation time was extended to 60 min, the current response become steady. Therefore, 40 min was chosen as the best incubation time for all the immunoassay.

### 3.4. Selectivity, Stability and Repeatability of the Immunosensor

**Figure 7:**
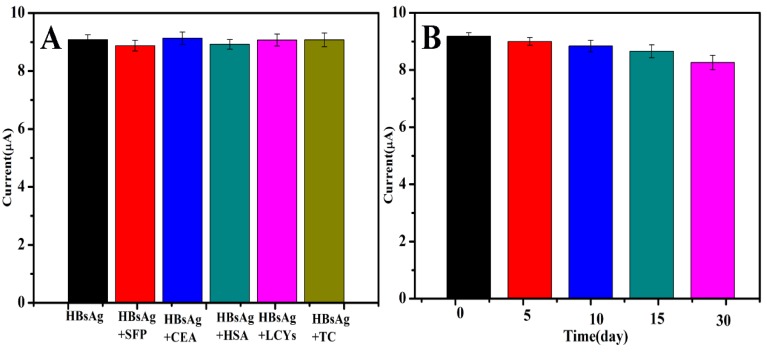
(**A**) Selectivity of the immunosensor to 100 ng/mL HBsAg, 100 ng/mL HBsAg + 100 ng/mL AFP, 100 ng/mL HBsAg + 100 ng/mL, 100 ng/mL HBsAg + 100 ng/mL HAS, 100 ng/mL HBsAg + 100 ng/mL LCYS and 100 ng/mL HBsAg + 100 ng/mL TC. (**B**) Stability of the immunosensor kept at 5, 10, 15 and 30 days in refrigerator at 4 °C.

To characterize the specificity of the immunosensor, the effect of possible interferences that might impact the determination of target analytes was investigated. DPV responses of the proposed immunosensor to 100 ng/mL of HBsAg containing different interferences, such as HSA, AFP, CEA, TC and LC, were assayed. Compared with the current response obtained by HBsAg only, variations from the interferents were less than 7%. The result indicated that the interference can be neglected, and the immunosensor has good selectivity for HBsAg (Figure 7A).

The stability of the immunosensor is a crucial factor in actual application and storage. The long-time stability of the immunosensor was studied by keeping the fabricated immunosensor in a refrigerator at 4 °C for 30 days when not in use. The peak current of the immunosensor was measured every five days in the first half of the month, and the current response still retained about 93% of the initial peak current, demonstrating that the immunosensor had good stability (Figure 7B).

Besides, the repeatability of the proposed immunosensor was studied. Five different immunosensors modified with the same procedures were evaluated with 100 ng/mL of HBsAg. The relative standard deviation (RSD) of the inter-assay was 2.8%, indicating that the immunosensor possessed good reproducibility.

### 3.5. Analytical Performance

**Figure 8:**
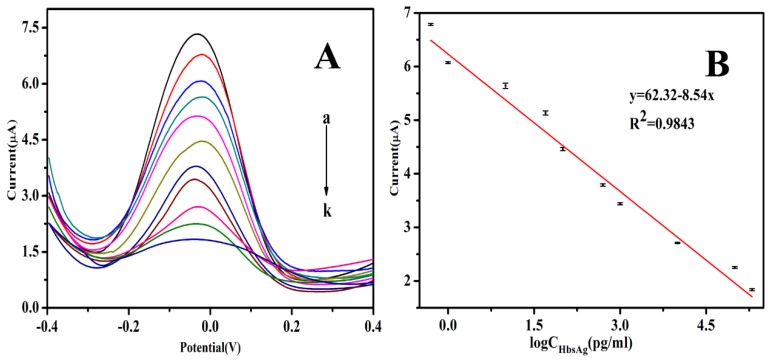
(**A**) The differential pulse voltammetry (DPV) response of the immunosensor after incubation with different concentration of HBsAg, from a to k: 0, 0.5 × 10^−3^, 1 × 10^−3^, 1 × 10^−2^, 5 × 10^−2^, 1 × 10^−1^, 5 × 10^−1^, 1, 10, 100 and 200 ng/mL in pH 7.4 PBS; (**B**) linear relationship between the current response and different concentration of HBsAg.

Under optimized detection conditions, the DPV responses of the immunosensor to different concentrations of HBsAg were obtained. Figure 8A shows that the peak currents of DPV decreased with an increased HBsAg concentration. The reason for this was that the formed immunocomplex on the electrode surface acted as an inert block layer, which hindered the electron transfer toward the electrode surface. As shown in Figure 8B, a linear relationship between the peak currents and the logarithmic values of HBsAg concentration was obtained in the range of 0.5 pg/mL to 200 ng/mL. The regression equation was y = 62.32−8.54x, with a correlation coefficient of 0.9843 and detection limit of 0.166 pg/mL (S/N = 3). The analytical performance of the immunoassay has been compared with the performances of other HBsAg immunoassays reported (Table 1), and the proposed immunosensor showed a widely linear range and a low detection limit.

### 3.6. Analysis of Real Samples

In order to investigate the reliability and accuracy of the label-free electrochemical immunosensor, five human serum samples were measured. The content of HBsAg in the serum samples was detected by the proposed immunosensor according to the relationship between the current response and HBsAg concentration. The obtained results were compared with those obtained by ECLIA, which was provided by the affiliated hospital of Guilin Medical College. As shown in Table 2, the relative errors between the two methods ranged from −2.83% to 14%, indicating that the fabricated immunosensor was suitable for real sample analysis. Therefore, the proposed immunosensor could be effectively applied in the quantitative detection of HBsAg in human serums and would have potential application in clinical diagnostics.

## 4. Conclusions

A novel, simple and label-free electrochemical immunosensor was developed for selective and sensitive detection of HBsAg. Thus, GO/Fe_3_O_4_/PB nanocomposites coated on SPE not only served as substrate materials for promoting electron transfer, but also acted as the electrochemical redox mediator. AuNPs that attached onto the modified electrode were used for HBsAb adsorption and further signal amplification. The proposed immunosensor showed excellent performance in the detection of HBsAg with a wide linear range, low detection limit, good biocompatibility, good selectivity and long-term stability. In summary, an ultrasensitive electrochemical immunosensor was developed for the detection of HBsAg. The simple and cost-effective sensing strategy provides a new promising platform for the design of a highly sensitive detection method, showing potential application for clinical immunoassays.

## Figures and Tables

**Table 1:** Comparison with other reported methods for the determination of HBsAg.

Immunosensor	Linear Range	Detection Limit (ng/mL)	Reference
Fe_3_O_4_@SiO_2_/MNPs/SELEX	1–200	0.1	[45]
Ab1@Ni AuPt-NGs/GCE	0.001–80	0.00031	[46]
GO-Fc-CS/Au NPs/GE	0.05–150	0.01	[47]
Nafion/gelatin/Au NPs/PDE	4–800	1.3	[48]
Chitosan-ferrocene/gold nanoparticles	0.5–305	0.016	[49]
EDC/NHS	5–3000	2.1	[50]
hemin-rGO/Au NPs	0.0001–1	0.00001	[51]
GO/Fe_3_O_4_/PB@AuNPs	0.0005–200	0.00016	This work

**Table 2:** Assay results of clinical serum samples using the proposed and reference methods.

Sample No.	Proposed Method (ng/mL) (*n* = 5)	Reference Method (ng/mL)	Relative Error (%)
1	5.7 × 10^−3^	5 × 10^−3^	14
2	0.121	0.118	2.54
3	9.27	9.4	1.38
4	98.21	96.35	1.94
5	125.96	129.63	−2.83
